# Massage in Simple Fractures of the Leg

**Published:** 1901-11-02

**Authors:** 


### MASSAGE IN SIMPLE FRACTURES OF THE LEG.

In the course of a long article on the treatment of fractures of the lower extremity Mr. Barnard,[1] of the London Hospital, describes the method adopted in applying massage to fractures of the leg. The main object of massage and passive movement is, he says, to prevent the formation of firm adhesions to the fracture. Massage may be commenced as soon as the fracture is set and comfortably supported on a back splint. It consists of simple stroking upward movements with the palm of the hand. Commencing with ten minutes a day it may be increased to half an hour at the end of three weeks. It removes extravasated blood effusion (as can be seen by the long streaks of staining which appear along the lymphatic channels of the thigh), soothes spasms, and maintains the circulation in the muscles. Passive movement may be commenced on the second or third day by lifting each toe independently off the splint on which the leg is firmly strapped. This produces movement in the deep flexors of the calf behind the seat of fracture. On the fourth day all the toes may be flexed together. On the fifth day the strap fixing the ankle should be removed, and whilst the ankle is firmly supported by one hand the ankle joint is gently flexed with the other. Each day after this the same routine is gone through—massage and passive movement of the toes, whilst the leg is fixed on the splint, and then passive movement of the ankle which is released for that purpose. On the tenth day the knee may be passively flexed by lifting it up from the splint, the fracture being supported by a kettle-holder splint strapped around it. In a fracture dealt with in this manner active movements may be commenced after the third week. They should be undertaken in the same order, namely, toes, ankle, and lastly knee. The advantages of treating fractures of the leg by massage and passive movements as here described are very considerable, tending as it does to hasten repair and to prevent stiffness of the joints, atrophy of muscles, obstructed circulation, and adhesions of tendons and nerves. The time which elapses before functional use is restored is said to be much reduced.

[1] The Practitioner, October.

